# Molecular Epidemiology, Seasonality and Phylogenetic Investigations of *Anaplasma ovis* in Small Ruminants from Diverse Agro-Climatic Regions of Punjab, Pakistan

**DOI:** 10.3390/microorganisms11102430

**Published:** 2023-09-28

**Authors:** Farhan Ahmad Atif, Sami Ullah, Raquel Cossío-Bayúgar, Muhammad Kashif, Aman Ullah Khan, Wen-Feng Wu

**Affiliations:** 1 Medicine Section, Department of Clinical Sciences, College of Veterinary and Animal Sciences, Jhang, Sub-Campus University of Veterinary and Animal Sciences, Lahore 54600, Pakistan; 2020-mphil-1111@uvas.edu.pk (S.U.); muhammad.kashif@uvas.edu.pk (M.K.); 2 Centro Nacional de Investigación Disciplinaria en Salud Animal e Inocuidad, Instituto Nacional de Investigaciones Forestales Agrícolas y Pecuarias (INIFAP), Carretera Federal Cuernavaca-Cuautla No. 8534, Jiutepec 62550, Morelos, Mexico; cossio.raquel@inifap.gob.mx; 3 Microbiology Section, Department of Pathobiology, College of Veterinary and Animal Sciences, Jhang, Sub-Campus University of Veterinary and Animal Sciences, Lahore 54600, Pakistan; amanullah.khan@uvas.edu.pk; 4 Department of Radiology, Ditmanson Medical Foundation Chia-Yi Christian Hospital, Chiayi 600, Taiwan

**Keywords:** *Anaplasma ovis*, phylogeny, sheep, goats, Pakistan

## Abstract

*Anaplasma (A.) ovis* is the most important cause of anaplasmosis in small ruminants. The current study was planned to estimate the molecular prevalence, risk factors, and phylogenetic analysis of *A. ovis* infection in sheep and goats from different agro-climatic regions of Central and Southern Punjab, Pakistan. A total of 400 jugular blood samples were collected from asymptomatic goats (*n* = 200) and sheep (*n* = 200) from the Jhang and Dera Ghazi Khan districts from January 2021 to February, 2023. Two hundred blood samples were collected from each district. Ten union councils (UC) were randomly chosen from each district, and 20 samples were collected from each UC based on the multistage cluster sampling technique. The samples were analyzed with PCR targeting the major surface protein (*msp4)* gene of *A. ovis*. The overall molecular prevalence of anaplasmosis was 57.5%. The disease occurrence was higher in Dera Ghazi Khan (61.5%) than in the Jhang district (53.5%). Infection positivity was greater in goats (65.5%) than in sheep (49.5%). Multivariate logistic regression analysis indicated that host species [sheep; Odds Ratio (OR) = 3.212; *p* = 0.000, Confidence Interval (CI) = 1.968–5.242], age (adult; OR = 2.606; *p* = 0.003, CI = 1.398–4.858), and acaricide use (never; OR = 13.671; *p* = 0.000, CI = 6.414–26.283) were significantly higher risk for *A. ovis* in small ruminants (*p* < 0.05; OR > 1). The sequencing and phylogenetic analysis of four representative isolates in the current study (Genbank numbers; Goats: OQ302202, OQ302203; Sheep: OQ319592, OQ319593) revealed novel strains of *A. ovis* with 97–100% similarity from different countries. The *msp4*-based goat isolates showed greater genetic diversity, while sheep genotypes showed homology with isolates from Italy, Spain, Hungary, Cyprus, Spain, Iran, and China. The current surveillance study will help in devising prevention and control strategies regarding anaplasmosis in small ruminants. However, there is a need for further study on the clinicopathological and vector competence aspects of these genotypes.

## 1. Introduction

Livestock is the principal subsector of agriculture, contributing 62.68% to agriculture and 14.36% to the national gross domestic product (GDP) of Pakistan in 2022–2023. Over eight million village families are associated with livestock production, deriving 35–40 percent of their income from livestock [1]. Pakistan is the third largest goat-producing country in the world, with a goat population of 84.7 million. Based on sheep production, Pakistan is the 12th largest country, with 32.3 million heads [1,2]. Livestock production is hampered by ticks and tick-borne diseases (TBD), which cause substantial economic losses associated with treatment, prevention, and control. Anaplasmosis is one of the major tick-transmitted diseases, and it has a remarkable impact on productivity, the health of small ruminants, and the livelihood of resource-limited farmers [3,4]. Anaplasma species that mainly infect small ruminants are *Anaplasma (A.) ovis*, *A. phagocytophilum*, and *A. marginale* [1,5]. *A. ovis* is an obligate intracellular intraerythrocytic Gram-negative bacterium that was first discovered in 1912 by Bevan [6]. It is the major cause of caprine/ovine anaplasmosis, and among other small ruminants worldwide, including in Pakistan, it is responsible for significant economic losses [3,7,8,9,10,11]. In Pakistan, recent studies have depicted the prevalence of *A. ovis* in small ruminants ranging from 12.5 to 59% [11,12,13,14,15,16].

The disease is usually subclinical in sheep and goats, but in acute cases, fever, severe anemia, decreased body weight, debility, decrease in milk production, pale mucous membranes, jaundice, abortion, and death may occur [5,17,18]. In addition, infection with *A. ovis* may predispose animals to contract additional infectious or parasitic diseases that worsen their health condition. After recovery from acute anaplasmosis, animals remain persistently infected and serve as reservoirs of infection, as well as a source for further spread [19,20]. A recent Spanish study demonstrated that untreated sheep remained permanently infected and served as carriers for their entire productive lives (4–6 years) after natural/experimentally infected cases [10].

Various biotic and abiotic factors, such as host (age, sex, breed, gestation, tick infestation, history of disease, body condition score, health status, carriers, and drug resistance), environment (climate, habitat, competent vector population, mechanical insect vectors, area, temperature, season, humidity, altitude, vegetation cover, rainfall, and reservoirs), and managemental determinants (animal movement, flock size, housing, floor, use of acaricide, grazing system, hygiene, stall feeding, agricultural and animal husbandry practices, and contaminated fomites), are associated with the occurrence of anaplasmosis [3,11,14,21,22]. The most important tick species known to transmit *A. ovis* are *Rhipicephalus* (*R.*) *sanguineus* (sensu lato), *R. bursa*, *R. pumilio*, *R. turanicus*, *Dermacentor* (*D.*) *marginatus*, *D. andersoni*, *D. nuttalli*, *Ixodes ricinus*, and *Hyalomma asiaticum* [23,24,25,26,27,28,29,30,31]. However, the vector competence for *A. ovis* is not known in Pakistan. The pathogen is transmitted by bites from hematophagous insects, transplacentally, and by blood-contaminated needles [8,32,33]. *A. ovis* also has zoonotic potential, and recent research has identified some variants among human patients from Iran, China, and Cyprus [32,34,35].

Anaplasmosis is usually diagnosed on the basis of a stained blood smear examination. This is quick to perform in clinical cases but unsuitable for detecting asymptomatic carriers with lower parasitemia. PCR based on nucleic acid detection provides much greater specificity and sensitivity than conventional blood smear microscopy [19]. Based on earlier reports, the *16S rRNA*, *msp4*, and *msp1a* genes have been used for the determination of the genetic diversity of *A. ovis* isolates [8,36,37]. The major surface proteins (MSPs) interact with the host and evolve more rapidly compared to other genes because they are subjected to selective pressure by the host immune system [25].

In Pakistan, limited epidemiological information on caprine and ovine anaplasmosis is available. There are only two reports from Pakistan that targeted the *msp4* gene for diagnosis. Among these, one study was performed in Northern Pakistan, and the second study depicted the prevalence of anaplasmosis only in sheep from one district with a small sample size [11,14]. The current study presents novel isolates during molecular detection, seasonality, and phylogenetic studies of *A. ovis* based on the *msp4* gene in both sheep and goats from two agro-ecologically diverse regions of Punjab, Pakistan.

## 2. Materials and Methods

### 2.1. Study Area and Sample Collection

**Figure 1:**
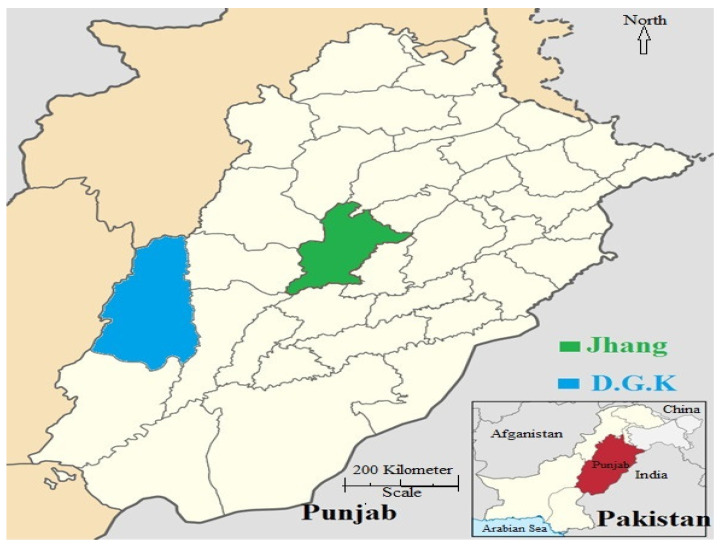
Map showing the two study districts. D.G.K.: Dera Ghazi Khan.

A total of 400 jugular blood samples were collected from asymptomatic sheep (*n* = 200) and goats (*n* = 200) from the Jhang and Dera Ghazi Khan districts of Punjab, Pakistan, from January 2021 to February, 2023. Two hundred blood samples were collected from each district. Ten union councils (UC) were selected from each district, and 20 samples were collected from each UC based on the multistage cluster sampling technique. These districts have diverse agro-climatic conditions, and the majority of people are greatly dependent on livestock, especially small ruminants. Jhang is located in the central part of the Punjab province between the Jehlum and Chenab rivers, situated at 31.27 latitude, 72.33 longitude, and 158 m elevation above sea level. Jhang has a warm (March, April, October, and November), sweltering (April to September), and humid climate, with extended summers. The average annual temperature during summer ranges from 55 to 70 °F (12.8–21.1 °C), while winter is cool, dry, and short, with an average temperature range from 42 to 55 °F (5.6–12.8 °C). Annual rainfall in the Jhang district is 0.89 inches (22.54 mm). The Dera Ghazi Khan district has a hot (February, March, April, October, and November), sweltering (April to September), and long humid summer, with an average high temperature range from 93 to 107 °F (33.9–41.7 °C), while winter is cool, dry, and short, with an average low temperature ranging from 47 to 59 °F (8.3–15 °C). Due to the barren mountains of Koh-e-Sulaiman and sandy soil, windstorms are common in summer. The average annual rainfall in the district was 0.04 inches (1.016 mm). It is located at 70.64 longitude and 30.04 latitude, with an elevation of 124 m above sea level [38,39] (Figure 1).

Jugular blood was collected aseptically and transferred to vacutainers containing EDTA. The samples were stored immediately in an icebox. All the samples collected from the sheep and goats were analyzed at the Post-Graduate Laboratory of Medicine, College of Veterinary and Animal Sciences, Jhang, Pakistan.

### 2.2. DNA Extraction

DNA was extracted from the blood samples using a Gene JET Whole Blood Genomic DNA Purification Mini Kit (Thermofisher Scientific, Waltham, MA, USA) following the guidelines of the manufacturer. Briefly, using a micropipette, a 200 μL blood sample was added to an Eppendorf tube, and then Proteinase K solution (20 μL) was added for protein destruction and release of nucleic acid. Later on, a lysis solution (400 μL) was added, and the product was subjected to vortexing. The sample was then incubated at 56 °C in a water bath for 10 min. Afterward, 200 μL of ethanol was added, and the mixture was shifted to a spin column and subjected to centrifugation at 6000× *g* for 1 min. The spin column was twice washed with 500 μL of wash buffer and centrifuged (8000× *g* for 1 min and 12,000× *g* for 3 min). In the last step, elution buffer (200 μL) was added, and the genomic DNA was collected after centrifugation (8000× *g* for 1 min). The purified DNA was stored at −20 °C in a microcentrifuge tube until used for further processing.

### 2.3. PCR

A set of primers was used to amplify the *msp4* gene sequence of *A. ovis* utilizing species-specific forward 5′TGAAGGGAGCGGGGTCATGGG3′ and reverse primers 5′GGTAATTGCAGCCAGGGACTCT3′ to yield a 347 base pair product, as described by Yousefi et al. [40]. A positive control in the form of a blood sample (2 mL) was obtained from Dr. Furhan Iqbal, Institute of Pure and Applied Biology, Bahauddin Zakariya University, Multan, Pakistan. The sample was collected from a PCR-positive goat from the Dera Ghazi Khan district, Pakistan. Distilled water was used as a negative control. PCR was conducted in a 20 µL reaction mixture containing 10 mm Tris-HCl (PH 9.0), 30 mM KCl, 1.5 mM MgCl_2_, 250 mm each dNTP, 0.5 mm each forward and reverse primer, 1 IU Taq DNA polymerase, and 2 µL of the DNA, and processed in an automated thermal cycler (T-100^TM^, BioRad, Hercules, CA, USA). Afterward, there was an initial denaturation step at 95 °C for 5 min. Each cycle consisted of denaturation at 94 °C for 30 s, annealing at 58 °C for 30 s, and extension at 60 °C for 30 s, followed by a final extension for 5 min. The PCR yields were examined on agarose gel 1.5% *w*/*v* with ethidium bromide (0.5 µg/µL of gel in 1× TAE buffer) using a DNA ladder of 50 bp (Thermofisher Scientific, Waltham, MA, USA; 00834919) connected to a power supply of 120 V for 30 min. To capture the gel image, the product was visualized using a UV illuminator (Biotech Fischer, Reiskirchen, Germany).

### 2.4. Estimation of Risk Factors

For the estimation of epidemiological risk factors, a pretested questionnaire was designed to collect information after consent regarding the biotic and abiotic risk factors of the area (Jhang, Dera Ghazi Khan), age (<6 months, 6–12 months, and >12 months), sex (male and female), breed (Kajli, Lohi, Buchi, Sipli, Beetal, Desi, Taddy, and Nachi), species (goat and sheep), tick infestation load (heavy > 50, moderate 10–50, and low 0–10 ticks), grazing system (free-grazing, semi-grazing, and zero/stall feeding), and acaricide use (regular, irregular, and never) associated with caprine and ovine anaplasmosis. The questionnaire, with close-ended questions, was completed on the spot at the time of blood sampling.

### 2.5. Sequencing and Phylogenetic Analysis

The positive representative samples (*n* = 4) were sent for nucleotide sequencing isolated from sheep and goats to Celemics Inc. using Barcode Tagged sequencing (BTSeq^TM^). The selected *msp4* sequences of *A. ovis* isolated from sheep, goat, sheep milk, roe deer, European red deer, humans, and ticks (*Rhipicephalus sanguineus* and *Dermacentor marginatus*) were utilized for the construction of a phylogenetic tree and compared with isolates from different countries (**Iran** LC430940, LC430942, MH017205, MH017206, MH411146, JQ621902, JQ621903, JQ663993, MH790273, MH790274, MK252270, KY091899, MK828060, MK828061; **India** MW561186; **Kenya** MF360026, MF360027, MF360028; **Tunisia** KY659323, KY659324, KC432643; **Turkey** KT251211, KY283958, MT344080, MT344081, MT344082, OM127900; **China** KU525114, KU525115, KU525120, KU525123, MZ502497, MZ502499, KU525117, KU525119, MZ502498, KU525113, KU525118; **Spain** HQ014384, EF067341; **Italy** AY702924, GQ130275; **Cyprus** FJ460455, FJ460443; **Pakistan** MT311200, MT311201, MT311202, MT311203; **Hungary** EF190509, EF190510, EF190511; **Egypt** MN882167, MN882168, OL859532, OL859533, P244843, OP244845, OP244846; and **Mongolia** LC141086). The evolutionary history was gathered using the maximum likelihood method and the Tamura 3-parameter model [41]. Evolutionary analyses were conducted in MEGA11 [42]. Statistical backing for the internal braces was set for bootstrap analysis with 500 replications. A *Rickettsia rickettsii* (U11021) sequence isolated from *Dermacentor andersoni* ticks was used as outgroup for phylogenetic analysis.

### 2.6. Statistical Analysis

Descriptive information related to risk factors was compared using the chi-square test. Univariable analysis was utilized to estimate the relationship between the molecular positivity of *A. ovis* and categorical variables. Variables with *p* < 0.20 were retained for the final multivariable logistic regression analysis for the estimation of linked risk factors using the Statistical Package for the Social Sciences software version 26.0 (IBM Corp., Armonk, NY, USA). A *p*-value less than or equal to 0.05 was considered statistically significant.

### 2.7. Ethical Approval

All procedures, including the handling and care of the animals, were performed as per the guidelines of the Institutional Review Committee for Biomedical Research, University of Veterinary and Animal Sciences, Lahore, Pakistan; approved vide letter No. DAS-623, dated 17 March 2022.

## 3. Results

### 3.1. Molecular Prevalence

**Figure 2:**
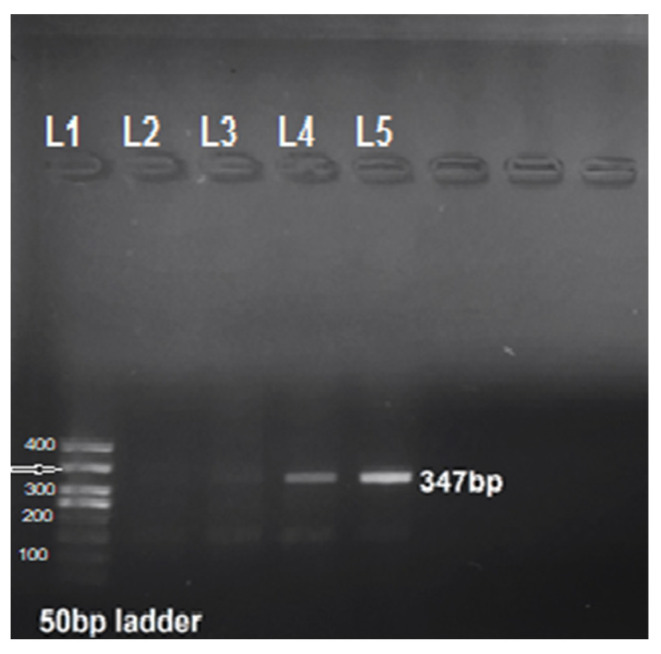
Gel image showing the 347 bp product of *A. ovis* in well number L5. Wells: L1 ladder; L2 and L3 were negative controls; L4 acted as a positive control.

For molecular prevalence, PCR was performed targeting the *msp4* gene using *A. ovis* species-specific primers. We noticed an overall prevalence of 57.5% (230/400). The PCR product of 347 bp was observed on the agarose gel using the UV gel illuminator (Figure 2). A higher prevalence was recorded in Dera Ghazi Khan (61.5%; 123/200) compared to Jhang (53.5%; 107/200). An equal number of blood samples were collected from sheep and goats. The results revealed a higher prevalence of anaplasmosis in goats (65.5%; 131/200) compared to sheep (49.5%; 99/200). Chi-square analysis indicated a statistically significant association between species-wise prevalence (*X*^2^ = 10.476, df = 1, *p =* 0.001). The results revealed that *A. ovis* was more common in adults than in young animals. On the basis of age, small ruminants were categorized into three groups (<6 months, 6–12 months, and >12 months). The prevalence was higher in the >12 months of age (64.9%; 133/205) than in the <6 months (40.5%; 30/74) and 6–12 months (55.4%; 67/121) age cohorts. A significant association was found among different age groups (*X*^2^ = 13.500, df = 2, *p =* 0.001). Sex-wise prevalence showed a significant association (*X*^2^ = 3.980, df = 1, *p =* 0.046), indicating a higher prevalence in females (61.7%) than in males (51.8%), irrespective of area and host type. For evaluating the role of tick infestation concerning pathogens, the animals were screened for tick infestation. Based on tick infestation load, the animals were categorized into three groups: low (0–10 ticks), moderate (10–50 ticks), and heavy (>50 ticks) tick infestations. In the region, the predominant tick species infesting small ruminants noticed were *Rhipicephalus sanguineus*, *Hyalomma anatolicum*, and *Hy. marginatum.* The animals infested with a higher number of ticks had higher infection rates than the tick-free animals, with a significant association (*X*^2^ = 9.514, df = 2, *p =* 0.009). To evaluate the prevalence based on grazing pattern, the animals were categorized into three groups: free-grazing, semi-grazing, and zero-grazing/stall-fed animals. The highest prevalence was noticed in the free-grazing system. Grazing had a significant effect on disease outcome (*X*^2^ = 16.917, df = 2, *p* = 0.000). A total of 59 goats and sheep were treated regularly, 99 were irregularly treated with acaricides, and 242 had never received any acaricidal treatment. The highest disease positivity was observed in the group that had never used acaricidal treatment, with a statistically significant effect (*X*^2^ = 68.048, df = 2, *p =* 0.000). The highest infection rate was recorded in the Nachi breed of goat. The breed factor showed as a significantly associated predictive variable (*X*^2^ = 68.048, df = 2, *p =* 0.000), while season had a non-significant effect on disease outcome.

### 3.2. Risk Factor Study

Risk factor estimation based on univariate regression analysis indicated that age (B = 0.504; OR = 1.655; *p* = 0.001, CI = 1.214–2.258), acaricide use (B = 1.317; OR = 3.734; *p* = 0.000, CI = 2.611–5.340), and sex (B = 0.534; OR = 1.705; *p* = 0.031, CI = 1.050–2.769) were significantly at a higher risk of acquiring *A. ovis* infection (*p* ≤ 0.05; OR > 1). Additionally, host species were retained for final multivariate analysis (B = 0.935; OR = 2.546; *p* = 0.070, CI = 0.925–7.007). Conversely, the independent variables of area, host species, tick infestation load, grazing pattern, breed, and season had non-significant effects on disease outcome (*p >* 0.05 and OR < 1), (Table 1).

Multivariate logistic regression analysis indicated that host species (goats; B = 1.167; OR = 3.212; *p* = 0.000, CI = 1.968–5.242), age (adult; B = 0.958; OR = 2.606; *p* = 0.003, CI = 1.398–4.858), and acaricide use (never; B = 2.615; OR = 13.671; *p* = 0.000, CI = 6.414–26.283) were significantly at higher risk for anaplasmosis (*p* ≤ 0.05 and OR > 1). Goats were 3.121 times at higher risk compared to sheep. Similarly, age-based variables demonstrated that animals of >12 months and 6–12 months of age had 2.606- and 1.702-fold higher chances of getting infected compared to <6 months of age, respectively. Likewise, small ruminants who never or irregularly treated with acaricide were at 13.671 and 2.986 times higher risk, respectively, compared to regular use of acaricide. Sex proved to be a non-significant risk factor, with a higher *p*-value (> 0.05) and lower odds ratio (<1) (Table 2).

### 3.3. Phylogenetic Analysis

**Figure 3:**
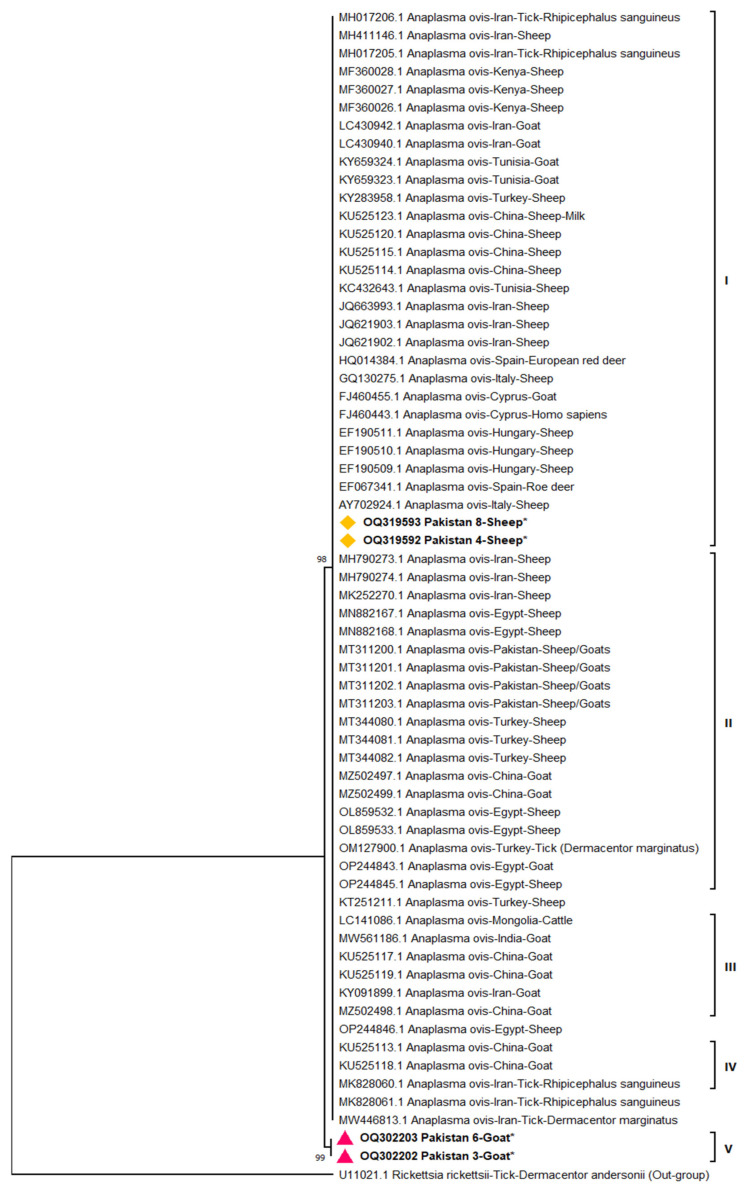
Maximum likelihood based evolutionary tree of Pakistani isolates of *A. ovis* (*msp4*) collected from sheep and goats. Sheep local isolates are indicated with yellow rhombus along with bold font and asterisk symbol *; goat local isolates are indicated in red triangle along with bold font and asterisk symbol *; I, II, III, IV are indicating clusters; V (clade).

Representative PCR-positive products (*n* = 4) were sent for nucleotide sequencing and later submitted to NCBI. We successfully obtained GenBank accession numbers for four isolates: two from sheep (OQ319592 and OQ319593) and two from goats (OQ302202 and OQ302203). The maximum likelihood method-based phylogenetic tree of *A. ovis* (*msp4*) indicated two major clades. Clade I was divided into four clusters. The current study’s isolates collected from sheep (OQ319592 and OQ319593) were in cluster I, along with genotypes of countries belonging to **Iran** MH017206, MH41146, MH017205, MF360028, MF360027, MF360026, LC430940, LC430942, KY659324, KY659323, KY283958; **Kenya** MF360026, MF360027, MF360028; **Tunisia** KY659323, KY659324, KC432643; **Turkey** KY283958; **China** KU525123, KU525120, KU525115, KU525114; **Spain** EF067341; **Italy** GQ130275; **Cyprus** FJ460443, FJ460455; and **Hungary** EF190509, EF190510, EF190511. Cluster II comprised of isolates from **Iran** MH790273, MH790274, MK252270; **Egypt** MN882167, MN882168, OL859532, OL859533, OP244843, OP244845; **Pakistan** MT311200, MT311201, MT311202, MT311203; Turkey MT344080, MT344081, MT344082, OM127900; and **China** MZ502497, MZ502499. Cluster III consisted of **Mongolia** LC141086; **India** MW561186; **China** KU525117, KU525119, MZ502498; and **Iran** KY091899. Cluster IV included isolates from China KU525113, KU525118 and Iran MK82860 (Figure 3). Our isolates from goats (OQ302202 and OQ302203) showed higher diversity and were grouped in a separate clade II. Our sequences from small ruminants showed 97–100% similarity with global isolates submitted to GenBank from different countries.

## 4. Discussion

Anaplasmosis is one of the most common tick-borne diseases (TBDs) worldwide, particularly in tropical and subtropical regions. *Anaplasma ovis* has been reported globally, including in Africa, Asia, Europe, and America [14,23,25,26,27,28,33,34,36,43]. In Pakistan, similar molecular studies are limited to two surveys that differ in terms of sample size, seasonality, targeted species, outcome, and study location [11,14]. In the current surveillance study, we estimated disease status based on molecular detection, risk factors, seasonality, and phylogenetic analysis of *A. ovis* (*msp4*) in both sheep and goats from the agro-ecologically diverse central and southern regions of Punjab, Pakistan.

The present study indicated an overall disease positivity of 57.5%. In Pakistan, earlier studies by Khan and coworkers are consistent with our research; they demonstrated a higher seropositivity of 59% in the Charsadda district, Pakistan, utilizing a commercially available competitive enzyme-linked immunosorbent assay [16]. The findings of our study strongly differ from the study by Niaz and his colleagues, who depicted a lower disease occurrence of 45.1% in Northern Pakistan [11]. Earlier reports from different countries depicted higher prevalence, ranging from 63 to 94%, *viz.* Iran, Iraq, Hungary, Turkey, Portugal, and Tunisia [3,20,26,44,45], while lower disease positivity rates compared to our study were documented from Senegal, Egypt, Slovakia, and India [36,46,47,48]. The diagnostic assay, local climatic conditions, flock size, poor hygienic conditions, and breeds may have been attributed to the variable occurrence of disease. A higher prevalence was recorded in Dera Ghazi Khan (61.5%; 123/200) compared to Jhang (53.5%; 107/200). The geographic location-based prevalence indicated a non-significant association. The higher occurrence of disease in Dera Ghazi Khan was due to higher vegetation cover, higher flock size, higher grazing, and poor husbandry practices.

The regression analysis indicated that goats were at a 3.212-fold higher risk compared to sheep. Similarly, the prevalence of anaplasmosis was significantly higher in goats than in sheep, regardless of the study area. These findings are in agreement with previous research by Selim et al. [22], Yousefi et al. [40], and Enkhtaivan et al. [49], as they demonstrated higher infection rates in goats (71.3%, 21.3%, and 34.7%, respectively) compared to sheep (69%, 18.3%, and 20.8%, respectively). There is a local trend of rearing goats with cows by resource-limited smallholder dairy farmers in Pakistan. This factor can contribute to a higher disease prevalence.

The results of the current study indicated a significant association between positive outcomes and the age of the animals. The highest prevalence (64.9%) was noticed in more than 12 months of age, while the lowest prevalence was in animals under 6 months of age. The age-wise results are consistent with Khan et al. [16] and Cabezas-Cruz et al. [50] and inconsistent with Hornok et al. [26]; they depicted a higher seroprevalence in adult small ruminants. The age-wise results are attributed to the fact that prevalence increases with age. The higher prevalence might be due to a higher number of carriers and adults, owing to exposure to various tick seasons [36,51].

The chi-square-based results of the current study on sex revealed a statistically significant association. The sex-wise results are supported by Khan et al. [16], who showed a 64.3% positivity rate in females for anaplasmosis in small ruminants. These findings are also endorsed by Niaz and colleagues, who depicted a higher disease positivity rate (16%) in females compared to males (7.3%) from Northern Pakistan [11]. The possible reason is that female animals are kept for a longer period of time and are likely exposed to several vector seasons.

The breed-wise prevalence in sheep expressed a significant relationship among different breeds, irrespective of age, sex, and area. However, the breed variable was not a significant determinant in univariate and multivariate regression analysis. These results are in agreement with Khan et al. [16], Cabzas-Cruz et al. [44], and Cabzas-Cruz et al. [50], who stated that the prevalence of TBDs is associated with breeds of domestic small ruminants, while Khan and colleagues depicted inconsistent findings in which breed was not a significant risk factor for acquiring anaplasmosis [51]. Certainly, local Pakistani breeds are more resistant to tick-borne infection. Regional Iranian breeds of sheep expressed a higher tendency to infection, parasitemia, and anemia during infection [52].

The highest prevalence was noticed in free-grazing animals (39.6%), followed by stall-fed or zero-grazing animals (36.5%), and the lowest in semi-grazing animals (23.9%). The chi-square test indicated a significant association between grazing pattern and disease in small ruminants, while regression analysis revealed a non-significant outcome. Yang and colleagues depicted that animals are more prone to infection, and the occurrence of anaplasmosis is associated with free-grazing [33]. Niaz and associates depicted that grazing is a predisposing factor for anaplasmosis [11]. Grazing could have increased contact between vectors, different farms, and other small animals [53].

A higher prevalence of *A. ovis* was recorded in the summer months. Our findings are consistent with those of Atif et al. [54], who also reported the highest prevalence of infection during summer, owing to a higher abundance and tick vector activity. Conversely, our findings are contradictory to those of Ashraf et al. [55], who mentioned that the highest occurrence was in autumn. When tick vectors are not widespread, it suggests that mechanical transmission might have played a role in the transmission of disease. This lets us suggest that the variations in disease prevalence rates are due to differences in geographical location and breeding microenvironment [11,14]. However, this factor was non-significant in all utilized statistical tests. The studied regions have extended dry, humid summers and short, cool winters, owing to less seasonal variation influencing disease occurrence.

In the current study, chi-square and regression analysis revealed that no application of acaricide is the most significant risk factor. The odds of developing the disease were 13.671 times higher in animals who had never received acaricide than in animals with regular acaricidal application. These results are in agreement with Rahman et al. [56], concluding that regular treatment with acaricide can control tick infestation, which eventually prevents anaplasmosis.

The phylogenetic analysis of the current study based on *msp4* revealed four novel isolates with a higher diversity of *A. ovis* genotypes in goats compared to sheep. The higher genetic diversity of goat isolates is linked to goat movement across the country for sale, purchase, and religious festivals, as we know that Pakistan is the third largest goat producer in the world. Our goat isolates were in a separate clade, while our sheep isolates were clustered with other geographical isolates from different countries like Italy, Spain, Hungary, Cyprus, Spain, Iran, and China. Our isolates from small ruminants showed 97–100% resemblance with isolates from different countries.

## 5. Conclusions

It is concluded that anaplasmosis is highly endemic in small ruminants. Never using acaricide is the major risk factor associated with the molecular occurrence of anaplasmosis. Phylogenetic analysis revealed novel isolates of *A. ovis* in sheep and goats. Further studies need to be conducted to evaluate the clinicopathological, economic loss, and vector transmission aspects of these genotypes for better prevention and control of anaplasmosis.

## Figures and Tables

**Table 1:** Univariate regression analysis for the estimation of risk factors related to anaplasmosis in sheep and goats.

Variable	β	*p*-Value	OR (95% CI)	Lower (CI)	Upper (CI)
Area	0.149	0.536	1.161	0.724	1.860
Species	0.935	0.070	2.546	0.925	7.007
Tick infestation load	−0.206	0.221	0.814	0.585	1.132
Age	0.504	0.001	1.655	1.214	2.258
Grazing pattern	−0.486	0.000	0.615	0.469	0.808
Acaricide use	1.317	0.000	3.734	2.611	5.340
Sex	0.534	0.031	1.705	1.050	2.769
Breed	0.020	0.860	1.020	0.818	1.272
Season	−0.098	0.443	0.906	0.705	1.165

OR = Odds ratio; CI = Confidence interval.

**Table 2:** Multivariate regression analysis for the estimation of risk factors related to anaplasmosis in sheep and goats.

Variable	Category	β	Wald	*p*-Value	OR (95% CI)	Lower (CI)	Upper (CI)
Species	Sheep *	--	--	--	--	--	--
	Goat	1.167	21.806	0.000	3.212	1.968	5.242
Age	<6 months *	--	9.504	0.009	--	--	--
	6–12 months	0.532	2.469	0.116	1.702	0.877	3.304
	>12 months	0.958	9.089	0.003	2.606	1.398	4.858
Acaricide use	Regular *	--	62.246	0.000	--	--	--
	Irregular	1.094	7.479	0.006	2.986	1.363	6.541
	Never	2.615	47.031	0.000	13.671	6.474	28.867
Sex	Male *	--	--	--	--	--	--
	Female	0.461	3.692	0.055	1.586	0.991	2.538

* Reference category.

## Data Availability

The data will be available upon request.
